# Long-term quantitative assessment of anti-SARS-CoV-2 spike protein immunogenicity (QUASI) after COVID-19 vaccination in older people living with HIV (PWH)

**DOI:** 10.1186/s12879-022-07737-0

**Published:** 2022-09-21

**Authors:** Jessica J. Tuan, Heidi Zapata, Lydia Barakat, Laurie Andrews, Anousheh Behnegar, Yee Won Kim, Jehanzeb Kayani, Suzana Mutic, Linda Ryall, Barbara Turcotte, Terese Critch-Gilfillan, Min Zhao, Syim Salahuddin, Shaili Gupta, Richard Sutton, Gerald Friedland, Brinda Emu, Onyema Ogbuagu

**Affiliations:** 1 Yale University School of Medicine, 333 Cedar Street, PO Box 208022, New Haven, CT 06510 USA; 2 Yale AIDS Program, Yale University School of Medicine, 135 College Street, Suite 323, New Haven, CT 06510 USA; 3 Yale Center for Clinical Investigation, Yale University School of Medicine, 2 Church Street South, Suite 401, New Haven, CT 06519 USA; 4 Veterans Affairs Connecticut Healthcare System, 950 Campbell Ave, West Haven, CT 06516 USA

**Keywords:** HIV, COVID-19, SARS-CoV-2, Immunogenicity, BNT162b2

## Abstract

**Background:**

The durability of immune responses to COVID-19 vaccines among older people living with HIV (PWH) is clinically important.

**Methods:**

We aimed to assess vaccine-induced humoral immunity and durability in older PWH (≥ 55 years, n = 26) over 6 months (post-initial BNT162b2 series). A secondary and exploratory objective was to assess T-cell response and BNT162b2 booster reactogenicity, respectively. Our Visit 1 (3 weeks post-initial BNT162b2 dose) SARS-CoV-2 humoral immunity results are previously reported; these subjects were recruited for Visit 2 [2 weeks (+ 1 week window) post-second vaccination] and Visit 3 [6 months (± 2 week window) post-initial vaccination] in a single-center longitudinal observational study. Twelve participants had paired Visit 2/3 SARS-CoV-2 Anti-Spike IgG data. At Visit 3, SARS-CoV-2 Anti-Spike IgG testing occurred, and 5 subjects underwent T-cell immune response evaluation. Thereafter, subjects were offered BNT162b2 booster (concurrent day outside our study) per US FDA/CDC guidance; reactogenicity was assessed. The primary study outcome was presence of detectable Visit 3 SARS-CoV-2 Anti-Spike-1-RBD IgG levels. Secondary and exploratory outcomes were T-cell immune response and BNT162b2 booster reactogenicity, respectively. Wilcoxon signed-rank tests analyzed median SARS-CoV-2 Anti-Spike IgG 6-month trends.

**Results:**

At Visit 3, 26 subjects underwent primary analysis with demographics noted: Median age 61 years; male n = 16 (62%), female n = 10 (38%); Black n = 13 (50%), White n = 13 (50%). Most subjects (n = 20, 77%) had suppressed HIV viremia on antiretroviral therapy, majority (n = 24, 92%) with CD4 > 200 cells/µL. At Visit 3, 26/26 (100%) had detectable Anti-Spike-1-RBD (≥ 0.8 U/mL). Among 12 subjects presenting to Visit 2/3, median SARS-CoV-2 Anti-Spike 1-RBD was 2087 U/mL at Visit 2, falling to 581.5 U/mL at Visit 3 (p = 0.0923), with a median 3.305-fold decrease over 6 months. Among subjects (n = 5) with 6-month T-cell responses measured, all had detectable cytokine-secreting anti-spike CD4 responses; 3 had detectable CD4 + Activation induced marker (AIM) + cells. Two had detectable cytokine-secreting CD8 responses, but all had positive CD8 + AIM + cells.

**Conclusions:**

Among older PWH, SARS-CoV-2 Anti-Spike IgG and virus-specific T-cell responses are present 6 months post-primary BNT162b2 vaccination, and although waning, suggest retention of some degree of long-term protective immunity.

## Background

Highly effective, novel mRNA vaccines were developed precipitously for prevention of 2019 Coronavirus disease (COVID-19), resulting in significantly decreased morbidity, mortality [1–3].

Key determinants of vaccine efficacy emerged following global COVID-19 vaccine rollout [4], including host factors (i.e. age, immunocompromised status), viral factors (i.e. variants of concern (VOC)/sub-variants exhibiting varying immune evasion levels), and vaccine-related factors (i.e. waning immune responses). These factors interact to cause increased susceptibility to SARS-CoV-2 infection/reinfection and have led to additional vaccine doses (boosters). However, key factors that should inform booster vaccine frequency are robustness, breadth, durability of immune responses to vaccination over time, correlated with clinical outcomes.

Incomplete information on COVID-19 vaccination durability in those with underlying immune dysregulation remains—particularly in people living with HIV (PWH). Therefore, we sought to assess level, breadth, durability of immune responses 6 months post-primary COVID-19 vaccination among older PWH.

## Methods

A cohort of PWH (≥ 55 years) who received BNT162b2 COVID-19 vaccination primary series at Yale New Haven Health System (YNHHS) vaccination sites were followed over 6 months. Individuals with prior laboratory-confirmed or breakthrough COVID-19 were excluded.

Subjects were recruited from a prepopulated schedule prior to visit/on-site for 3 visits: Visit 1 [3 weeks post-first vaccination (published previously [5])]; Visit 2 [2 weeks (+ 1 week window) post-second vaccination]; Visit 3 [6 months (± 2 week window) post-first vaccination].

SARS-CoV-2 semi-quantitative Anti-Spike 1-RBD IgG was performed (Roche Elecsys, under US FDA Emergency Use Authorization [99.5% sensitivity, 99.8% specificity]) on cryopreserved sera (Visit 2), and fresh sera (Visit 3) to determine Visit 2/3 antibody levels. Positive SARS-CoV-2 qualitative anti-nucleocapsid antibody (Roche Elecsys) led to exclusion of subjects with COVID-19 history from analyses.

### SARS-CoV-2 vaccine T-cell immunogenicity testing

Cryopreserved PBMCs were thawed, rested, and cultured (6-h) in SARS-CoV-2 peptide pool (1 μg/ml, Miltenyi Biotec), then stained for intracellular cytokine stating (ICS) assay, and co-stimulated with anti-CD28/anti-CD49d for activation induced marker (AIM) assay. Antibodies (Biolegend): anti-CD3 (UCHT1), anti-CD4 (SK3), anti-TNF-α (MAb11), anti-OX40 (Ber-ACT35), anti-CD137 (4B4-1), anti-CD69 (FN50, Biolegend); Antibodies (BD Biosciences): anti-CD8 (SK1), anti-IFN-γ (B27). Flow cytometry data was acquired on LSRFortessa and analyzed by FlowJo v.10.8.0.

### Data collection

Electronic medical record review yielded subject demographics, body mass index (BMI), co-morbidities including immunosuppressed status, HIV history (duration, antiretroviral therapy (ART), recent CD4, viral load).

### Statistical analysis

Data distribution was non-Gaussian; thus, non-parametric paired analysis (Wilcoxon signed-rank test) using Stata (v16.1) compared Visit 2/3 antibody levels. Statistical significance was determined at p-value < 0.05.

### Ethical approval

This study received Yale Human Investigations Committee and Institutional Review Board approval (HIC # 200030266) and written informed consent from subjects was obtained.

## Results

**Fig. 1:**
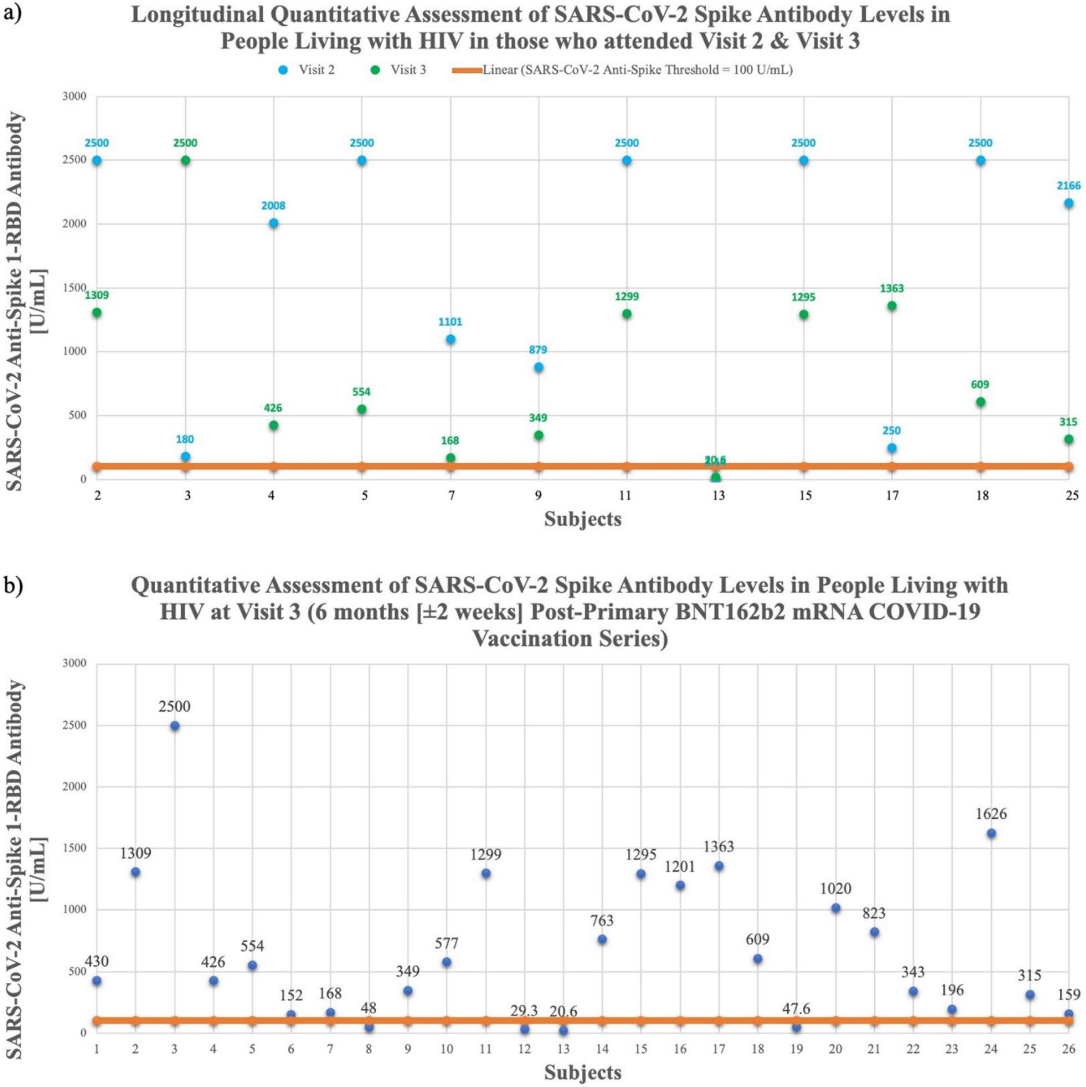
**a** Quantitative assessment of SARS-CoV-2 Spike Antibody levels in people living with HIV who attended Visit 2 (2 weeks [+1 week window) post-second BNT162b2 COVID-19 vaccination series) and Visit 3 (6 months [± 2 weeks] post-primary BNT162b2 COVID-19 vaccination series) (n= 12). Two patients had significantly higher antibody levels at Visit 3, compared to Visit 2. **b** Quantitative assessment of SARS-CoV-2 Spike Antibody levels in people living with HIV (n= 26) at Visit 3 (6 months [± 2 weeks] post-primary BNT162b2 COVID-19 vaccination series)

Thirty-one met inclusion criteria (5 excluded [COVID-19 history (n = 3), pre-Visit 3 booster recipients (n = 2)]). Twenty-six were included in primary analysis (Demographics, Co-morbidities, SARS-CoV-2 antibody results in Table 1). All took ART, majority (n = 24, 92%) had CD4 > 200 cells/µL, and 20/26 were virologically suppressed; 6 had detectable viremia (< 100 copies/mL). All 26 participants (100%) had detectable Visit 3 Anti-Spike-1-RBD IgG [reference < 0.8 U/mL] (Fig. 1/Table 1). In a subset participating in both Visits 2 and 3 (n = 12) median SARS-CoV-2 Anti-Spike 1-RBD was 2087 U/mL (n = 12) at Visit 2, which fell to 581.5 U/mL (n = 12) at Visit 3 (p = 0.0923), reflecting a 6-month median 3.305-fold decrease (Fig. 1a) though not statistically significant. Median SARS-CoV-2 Anti-Spike 1-RBD for all Visit 3 subjects (n = 26) was 492 U/mL (Fig. 1b). Using a clinical correlate of Anti-Spike-1-RBD antibody ≥ 100 U/mL as a disease protection threshold [6], 22/26 (84.6%) met positivity criterion. Four subjects were sub-threshold: One had chronic kidney disease (CKD); 3 had multiple co-morbidities, including heart transplant on tacrolimus (1), CKD (1), and morbid obesity (1) (Table 1).

**Table 1:** Participant demographics, co-morbidities, and SARS-CoV-2 antibody results

Subject	Visit 2 Quantitative SARS-CoV-2 Anti-Spike 1-RBD Antibody [U/mL] (N = 12) (Subjects who attended Visit 2 & 3) Reference range [< 0.8 U/mL]	Visit 3 Quantitative SARS-CoV-2 Anti-Spike 1-RBD Antibody [U/mL] (N = 26) (Total subjects who attended Visit 3) Reference range [< 0.8 U/mL]	T-cell Immunity Subset	Age (Visit 1) [Years]	Gender	Race	Ethnicity	CD4 count [cells/µL]	HIV Viral Load [copies/mL]	Body Mass Index [kg/m^2^]	Co-morbidities
1		430		68	Male	White	Non-Hispanic	553	0	22.38	Heart Disease, Substance Use Disorder
2	2500	1309		56	Male	White	Non-Hispanic	1123	0	29.09	History of Cancer, Heart Disease, Lung Disease, Overweight
3	180	2500		50	Male	White	Non-Hispanic	407	0	25.04	History of Cancer, Stroke, Advanced Lung Disease, Smoking history
4	2008	426	T-Cell Subset	63	Male	Black	Non-Hispanic	176	0	29.68	
5	2500	554		55	Male	White	Hispanic	1242	0	27.17	Lung Disease, Substance Use Disorder
6		152		63	Female	Black	Non-Hispanic	374	0	20.66	Smoking History
7	1101	168		64	Male	White	Non-Hispanic	900	0	27.2	Substance Use Disorder
8		48		61	Male	White	Non-Hispanic	801	0	28.86	Chronic Kidney Disease
9	879	349		61	Female	Black	Non-Hispanic	984	0	24.2	
10		577		80	Male	Black	Non-Hispanic	718	0	27.25	
11	2500	1299		55	Female	White	Non-Hispanic	359	0	26.63	
12*		29.3		66	Male	Black	Non-Hispanic	339	32.9	26.8	Chronic Kidney Disease, Diabetes Mellitus, Heart Disease, Lung Disease
13	2.14	20.6		66	Female	Black	Non-Hispanic	103	27.5	25.16	Heart transplant recipient (on tacrolimus), Diabetes Mellitus, Heart Disease, Stroke
14		763		57	Male	Black	Non-Hispanic	1078	0	31.67	Lung Disease
15	2500	1295		63	Female	Black	Non-Hispanic	616	38	24.08	
16		1201		56	Male	White	Non-Hispanic	518	0	33.47	Lung Disease
17	250	1363		61	Male	White	Non-Hispanic	729	36.7	33.73	Other Cardiovascular Disease, Alcohol use, Substance Use Disorder
18	2500	609		58	Male	White	Non-Hispanic	720	0	35.89	Other Cardiovascular Disease, Alcohol use
19		47.6		60	Male	Black	Non-Hispanic	786	99.7	47.9	Advanced Liver Disease, Diabetes Mellitus, Heart Disease, Other Cardiovascular Disease, Lung Disease
20		1020	T-Cell Subset	65	Female	Black	Non-Hispanic	539	0	27.44	Advanced Liver Disease, History of Cancer, Smoking History, Substance Use Disorder
21		823		61	Male	Black	Non-Hispanic	746	0	29.42	History of Cancer, Heart Disease, Substance Use Disorder
22		343	T-Cell Subset	62	Female	White	Hispanic	706	50.6	30.21	History of Cancer, Lung Disease, Smoking History
23		196	T-Cell Subset	58	Female	White	Non-Hispanic	1413	0	42.91	Smoking History
24		1626		64	Male	Black	Non-Hispanic	612	0	38.72	Advanced Liver Disease, History of Cancer, Active Cancer, Diabetes Mellitus
25	2166	315	T-Cell Subset	60	Female	Black	Non-Hispanic	225	0	39.49	Advanced Liver Disease, History of Cancer, Other Cardiovascular Disease, Alcohol Use
26		159		56	Female	Black	Non-Hispanic	898	0	24.26	Smoking history
Median value	2087	492									

**Fig. 2:**
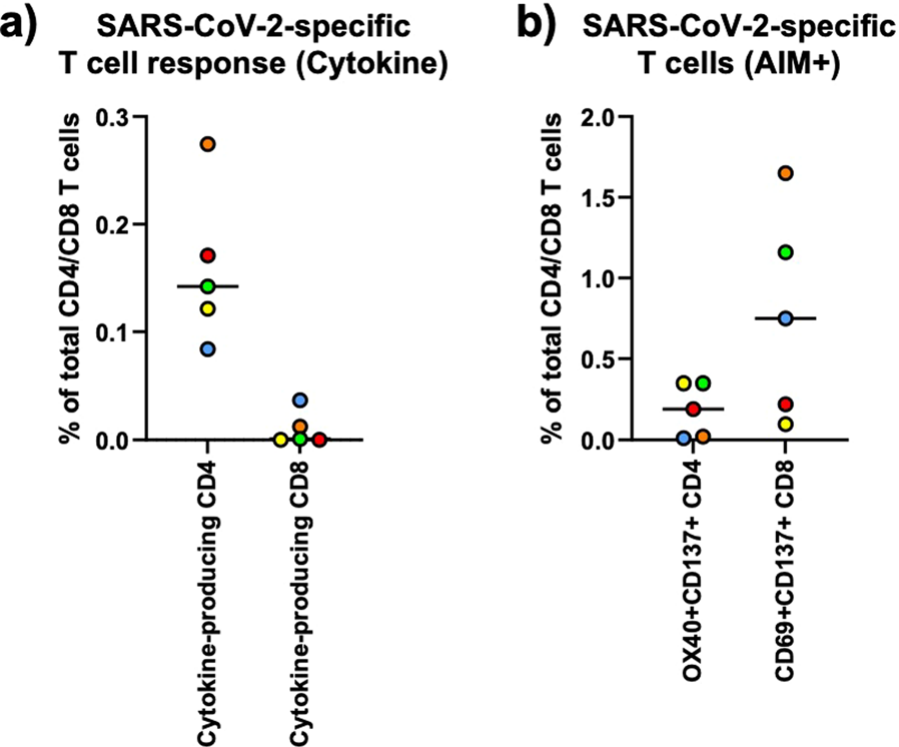
Immunologic T cell subset testing. **a** SARS-CoV-2-specific T cell response after intracellular cytokine staining assay (ICS, 6 h). Cytokine production was defined as IFγ+TNFα–, IFγ+TNFα+ and IFγ-TNFα+ combined. Cytokine production was measured within live CD3+CD4+CD8- cells for CD4 response and live CD3+CD4-CD8+ cells for CD8 response. **b** SARS-CoV-2-specific T cells after activation induced marker assay (AIM, 20 h). SARS-CoV-2-specific CD4 T cells and CD8 T cells were defined as live CD3+CD4+CD8-OX40+CD137+ cells and CD3+CD4+CD8-CD69+CD137+ cells, respectively

**Fig. 3:**
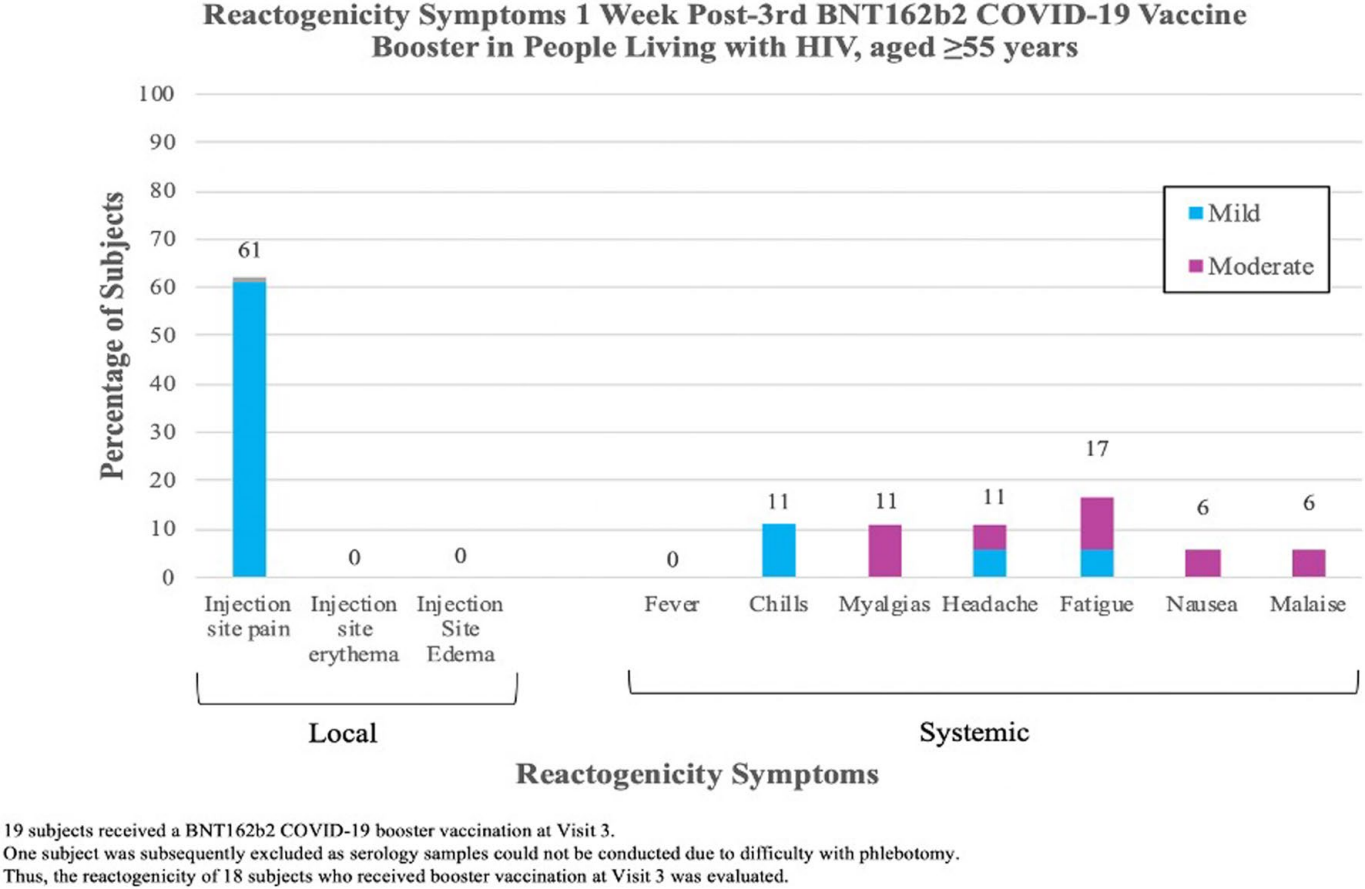
Reactogenicity symptoms 1-week post-3rd BNT162b2 COVID-19 vaccine booster in People living with HIV, aged ≥55 years

Eighteen Visit 3 subjects receiving BNT162b2 booster had reactogenicity evaluated 1-week post-booster. All subjects (100%) reported ≥ 1 mild-moderate symptom (Fig. 3): Injection site pain 61% (n = 11); fatigue 17% (n = 3); chills, headaches, or myalgias 11% (n = 2); nausea or malaise 6% (n = 1). Among these subjects, a cohort (n = 5) had T-cell immunologic responses analyzed (Fig. 2). All (n = 5) had detectable cytokine-secreting anti-spike CD4 responses; 3 had detectable CD4 + AIM + cells. Two had detectable cytokine-secreting CD8 responses, but all (n = 5) had positive CD8 + AIM + cells.

## Discussion

Our study results demonstrate there are detectable circulating anti-spike RBD antibodies 6-months post-primary COVID-19 BNT162b2 vaccination series in older PWH. Guidelines for PWH have described older PWH as people who are 50 years of age or older [7]. Using a threshold of Anti-Spike-1-RBD antibody ≥ 100 U/mL as a correlate of COVID-19 protection [6], it is remarkable that 84.6% of subjects met 6-month threshold criterion. Of note, there is limited data regarding the clinical applicability of using this Elecsys Anti-SARS-CoV-2 S RBD assay and the implications of its semi-quantitative antibody levels as it relates to the degree of immunity or protection against COVID-19 in vaccinated individuals [8]. Our cohort had significant variability and rather broad range of Anti-Spike-1-RBD levels, which may reflect participant characteristics, co-morbidities influencing vaccine responses. Four subjects below the clinical correlate of protection had multiple co-morbidities, including CKD in the majority, and 1 heart transplant recipient on tacrolimus. Our cohort, though older, were virologically suppressed, most with CD4 > 200 cells/µL. Thus, underlying HIV may not have negatively influenced vaccine responses, unlike those with lower CD4 counts, as observed in other studies.

We found significant circulating antibody waning over time, as observed in other cohorts. Waning immunity has been associated with clinical endpoints of increased vulnerability to SARS-CoV-2 infection/reinfection, particularly where circulating VOC demonstrate significant immune evasion. Thresholds at which these events occur must be well-defined. Thus, it is important to correlate immune responses (including qualitative/quantitative) with clinical outcomes, among different populations/hosts, to inform immunologic assessment, clinical significance—and importantly—vaccine booster frequency.

Much attention has been given to assessing cell-mediated immune responses post-COVID-19 vaccination. While circulating neutralizing antibodies emerged as primary correlate of protection against infection, memory B- and T-cells—which modulate adaptive immune responses, acting as effector cells—serve as secondary lines of defense against disease progression and severity following SARS-CoV-2 infection and may exhibit greater durability [9]. Though T-cell responses were assessed in a small cohort, the robust persistence of SARS-CoV-2 Spike-specific and functional T-cells 6-months post-primary mRNA vaccination among older PWH is encouraging, warranting exploration. Spike-specific T-cells generated by BNT162b2 exhibit wide breadth and retain activity against emerging VOC [10], although their immunoprotective role is not well-defined.

Regarding reactogenicity, booster vaccine was well-tolerated. Most experienced local injection site pain with limited systemic reactogenicity, on par with other booster dose studies [11].

Our study has important limitations. Our single academic center cohort comprised PWH ≥ 55 years with well-controlled HIV, robust CD4 counts, which may not represent HIV-infected cohorts with dissimilarities and a younger cohort of people living with HIV [12]. However, it does provide important insight about older PWH, a demographic increasing annually as majority of US PWH are ≥ 50 years [7]. We evaluated response to a specific mRNA vaccine, so findings may not extrapolate to other (mRNA) vaccines/platforms. Although we lacked an HIV-uninfected control group, immune responses published among other cohorts provide context for interpreting our data. Notwithstanding, the absence of standardized antibody assays remains challenging for direct study result comparison. Thus, a more standardized method of assessing SARS-CoV-2 humoral immunity and correlates of immune protection is needed; ongoing research is being conducted to establish international standards to interpret humoral immunity results using different testing platforms and units of measurement [8]. We excluded participants with prior or breakthrough COVID-19, so as not to bias immunologic assessments, which may inadvertently select for more optimal vaccine responses.

## Conclusions

Our prior data highlighted the importance of 2-dose COVID-19 primary vaccination series in older PWH [5]. This study demonstrates that though there is waning immunity by 3.305-fold over 6-months post-primary COVID-19 vaccination, there is a degree of retention of humoral immunity among most older PLWH. A sub-study revealed presence of Spike-specific T-cell responses. Our findings suggest that older PWH retain immunologic benefit from vaccination 6-months post-vaccination, though booster doses are needed to maintain optimal antibody levels over time.

## Data Availability

The data that support the findings of this study are available but protected under Institutional Review Board at Yale given the sensitive nature of patient health information and, thus, restrictions apply to the availability of these data, which were used under license for the current study, and so are not publicly available. Data are however available from the authors upon reasonable request and with permission of Yale.
